# Editorial: Carbon-based materials: powering the future of energy and environmental progress

**DOI:** 10.3389/fchem.2024.1481960

**Published:** 2024-10-07

**Authors:** Nimra Nadeem, Muhammad Zahid, Qamar Abbas, Asim Jilani

**Affiliations:** ^1^ Department of Chemistry, University of Agriculture Faisalabad, Faisalabad, Pakistan; ^2^ Institute for Chemistry and Technology of Materials, Graz University of Technology, Graz, Austria; ^3^ Center of Nanotechnology, King Abdulaziz University, Jeddah, Saudi Arabia

**Keywords:** carbon-based composites, energy storage, environmental remediations, MXenes based composites, smart textile

The careful selection of the title for this Research Topic is well justified. Carbon materials play significant role in improving the quality of human lives encompassing a wide range of applications including energy storage, photovoltaics, catalysis, sensors, water purification, and other environmental and scientific disciplines. These fields drive the developments of new technologies characteristic of the 4th Industrial Revolution. In this Research Topic on “*Carbon-based materials: Powering the Future of Energy and Environmental Progress*” published in the Frontiers of Chemistry, salient aspects of carbon-based materials have been covered with six articles coming from internationally reputed scientists.

- The paper on energy storage from Supiyeva et al. from Kazakhstan present the use of **eco-friendly aqueous electrolyte** used for developing supercapacitor that can operate in a wide temperature window of −40°C to + 60°C. The main idea is to present a next-generation solution for fast energy storage in nanoporous carbon electrodes, which is low-cost and safe. The porous carbon network allows for maintaining the liquid state of bulk electrolytes and prevents them from freezing. Additionally, the hydrogen evolution in aqueous electrolyte due to electroreduction of water is altered by the additional of methanol, and all this fine-tuning of electrolyte leads to fully operating supercapacitor at low and high temperatures.
- In the area of smart textiles, Albargi et al. developed the novel piezoresistive strain sensor using a graphene/siloxane composite conductive ink applied to plied auxetic yarn (PAY), marking a significant advancement in wearable sensor technology. The blend of lycra and cotton yarns, braided core-spun and coated with siloxane polymer resin, improves mechanical durability and longevity. The innovative design enhances the negative Poisson’s ratio, resulting in a 2.5 times increase in sensitivity and a fivefold increase in strain range. This sensor efficiently translates mechanical strain into electrical resistance changes, offering promising health monitoring and structural diagnostics applications, heralding a new era of flexible, textile-based wearable technologies.
- In the domain of environmental protection, Shahid et al. presented the use of IoT-based sensor networks for enhancing air quality monitoring in the Middle East. The study provides the real-time, accurate data for tracking pollution trends and informing decisions. These networks, especially carbon-based sensors, offer extensive spatial coverage and are crucial for detecting gaseous pollutants. They are vital in mitigating health risks, protecting ecosystems, and addressing climate change. By integrating IoT-based systems with advanced data analysis techniques like machine learning and AI, policymakers can design effective mitigation strategies, aligning with Sustainable Development Goals and promoting sustainable development in resource-constrained regions.
- In the field of biomedical applications of advanced nanomaterials, Shahbaz et al. successfully synthesized high-purity chromium aluminum carbide via facile sol-gel method. Comprehensive characterization confirmed their structural, morphological, and chemical properties, making them suitable precursors for MXenes production. Notably, the synthesized phases exhibited significant antifungal activity against *Candida* albicans and minimal impact on the viability of *E. coli* and *S. aureus*. Cytotoxicity studies revealed that Cr_2_AlC induced oxidative stress in HepG2 cells, while Cr_3_AlC_2_ and Cr_4_AlC_3_ showed lower cytotoxicity, underscoring their potential for biomedical applications. This research highlights the versatile applications of MAX phases in materials science and biomedicine.
- In the domain of environmental remediations, Alkorbi et al. reported the management of industrial dye effluents to reduce its significant environmental risks. The authors investigate the photocatalytic degradation of Direct Red 28 dye using a ternary composite of graphitic carbon nitride, TiO_2_, and polyorthoanisidine (g-C_3_N_4_/TiO_2_/POA), synthesized via *in situ* oxidative polymerization of o-anisidine. The detailed characterization of composite material ensured the successful fabrication. Photocatalytic tests demonstrated 86% degradation of Direct Red 28 at 30 mg/L dosage in 240 min at pH 2, with a 79% degradation rate after four cycles, highlighting the composite’s stability and efficiency. The enhanced reusability and effectiveness are attributed to increased light absorption and reduced electron-hole recombination in the presence of g-C_3_N_4_ and POA.
- Zainab et al. empowered the field of self-cleaning textiles in a study by synthesizing superhydrophobic materials through an eco-friendly, scalable, one-step hydrothermal process. The resulting superhydrophobic carbon quantum dots SCQDs exhibited crystalline carbon cores and amorphous hydrophobic polymeric chains, with a size distribution of 2–8 nm and an average size of 5 nm, produced by microwave-assisted carbonization. Their ultra-small size and high nanoscale surface roughness enabled water droplets to roll off, achieving a contact angle 163.64°. Applied to textile fabric via dip coating with a polydimethylsiloxane (PDMS) binder, SCQDs displayed aggregation-induced emission under UV light due to stable passivated surface states. The coated fabric maintained self-cleaning properties at a 10° angle without compromising porosity or comfort. A solvent-induced precipitation and filtration method for SCQD aggregates offered scalability over conventional synthesis methods. This facile approach for fabricating SCQD-coated surfaces demonstrates significant potential for commercial-scale self-cleaning applications in healthcare and outdoor wearables, maintaining superhydrophobicity in harsh environments without secondary pollution.

